# Impacts of human activities on the supply of marine ecosystem services: A conceptual model for offshore wind farms to aid quantitative assessments

**DOI:** 10.1016/j.heliyon.2023.e13589

**Published:** 2023-02-15

**Authors:** Lennert Van de Pol, Katrien Van der Biest, Sue Ellen Taelman, Laura De Luca Peña, Gert Everaert, Simon Hernandez, Fiona Culhane, Angel Borja, Johanna J. Heymans, Gert Van Hoey, Jan Vanaverbeke, Patrick Meire

**Affiliations:** a ECOSPHERE Research Group, University of Antwerp, Universiteitsplein 1, 2610 Wilrijk, Belgium; b Ghent University, Green Chemistry and Technology, STEN Research Group, Coupure Links 653, 9000 Ghent, Belgium; c Flanders Marine Institute, Wandelaarkaai 7, B8400 Ostend, Belgium; d Ghent University, GhEnToxLab, Coupure Links 653, 9000 Ghent, Belgium; e School of Biological and Marine Science, University of Plymouth, Devon PL4 8AA Plymouth, United Kingdom; f AZTI, Marine Research, Basque Research and Technology Alliance (BRTA), Herrera Kaia, Portualdea s/n, 20110 Pasaia, Spain; g European Marine Board, Jacobsenstraat 1, 8400 Oostende, Belgium; h Scottish Association for Marine Science, Scottish Marine Institute, Oban, United Kingdom; i Flanders Research Institute of Agriculture, Fishery and Food, Jacobsenstraat 1, 8400 Oostende, Belgium; j Royal Belgian Institute for Natural Science, Operational Directorate Natural Environment, Vautierstraat 29, 1000, Brussels, Belgium

**Keywords:** Marine ecosystem services, Impact assessment, Offshore wind energy, Conceptual model, Ecosystem functioning, Indicators

## Abstract

Increased pressures from human activities may cause cumulative ecological effects on marine ecosystems. Increasingly, the study of ecosystem services is applied in the marine environment to assess the full effects of human activities on the ecosystem and on the benefits it provides. However, in the marine environment, such integrated studies have yet to move from qualitative and score-based to fully quantitative assessments. To bridge this gap, this study proposed a 4-tiered method for summarizing available knowledge and modelling tools to aid in quantitative assessments of ecosystem services supply. First, the ecosystem functioning mechanisms underlying the supply of services are conceptually mapped. Second, the impacts of the human activity of interest are summarized and linked to the first conceptual model in a case-specific model of ecosystem services supply. Third, indicators are selected that would best represent changes in the most important parameters of the conceptual model in a quantitative manner. Fourth, the knowledge gained in the previous steps is used to select models that are most useful to quantify changes in ecosystem services supply under the human pressure of interest. This approach was applied to the case study of offshore wind energy in the Belgian part of the North Sea, which is one of the most rapidly expanding industries in the marine environment globally. This study provides a useful tool to proceed towards quantification of marine ecosystem services, highlighting the need for a fully integrated approach to developing environmental impact assessment tools.

## Graphical abstract

**Figure:**
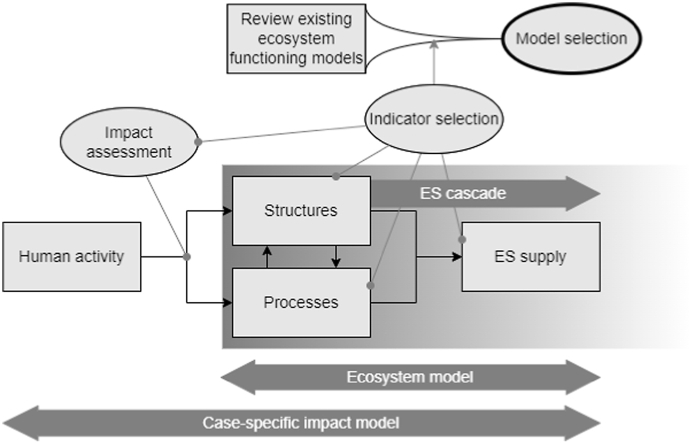

## 1 Introduction

Increasing human activity at sea such as offshore wind energy and aquaculture, on top of ‘traditional’ industries such as fisheries and transport, may put more pressure on ecosystems, causing cumulative ecological effects and risk of losing ecosystem services (ES) [1]. According to the rationale of the ES cascade framework, services are supplied by the ecosystem's structures and processes and the interactions between them [2]. These services subsequently provide certain societal benefits, which can be valued by different stakeholders [3]. Human activities can unintentionally alter (positively and/or negatively) the benefits society gains from the marine environment through changes in the underlying ecosystem structures and processes [3]. There is increasing interest for taking ES into account when managing natural resources, because it can help identify hidden trade-offs or additional benefits of human interactions with the natural environment [4,5]. Applying an ES-based approach will help increase the sustainability of human activities at sea that depend on natural resources, safeguarding the supply of ES and thus supporting marine and maritime sectors in the long term (conform Sustainable Blue Economy [6]).

In the marine environment, Ecosystem Services Assessments (ESA) have not been as advanced and detailed as in the terrestrial environment [[7], [8], [9], [10], [11]] and assessment methods are less widely available [5,8,12]. This is because there is often a lack of data [13] and impacts of human activities at sea are not as visible as those on land [10]. Hence, the full impacts of human activities on the ecosystem and the services it provides are often not well known. This is why marine ES assessments are often assessed using expert-based, qualitative approaches or the benefit-transfer method in which ES of a site are quantified by applying values from other sites [14]. However, these approaches do not fully take into account the ecological processes that drive the delivery of ES, including the dynamic interactions between ecosystem structures, species, ecosystem processes and humans [15]. Only by identifying complete cause-effect chains will it be possible to understand which processes underly changes in ES, and to determine where in the system interventions should take place to optimize the supply of ES.

The widely applied Driver-Pressure-State-Impact-Responses (DPSIR) framework [16] or derivatives thereof [17] have been used as a conceptual framework to unravel the complete cause-effect chain from human activities to changes in ecosystem structures and processes and benefits to society. Among others, DPSIR frameworks have been applied to manage marine ES [18,19], implement European Directives [20], perform environmental risk assessment [21] and develop management tools [22]. However, quantification of the links represented in existing conceptual models is still largely lacking [23]. Many marine ecosystem models exist to quantify changes along (parts of) these cause-effect chains [24]. But the extent to which they are suitable for ES assessments varies, depending on the characteristics of the models (the parameters and processes they are able to capture), their capacity to link with other models representing different parts of cause-effect chains, and the purpose of the assessment and the user of the model (e.g. complexity, economic valuation, …). In order to take this step towards quantification, an essential task is to get an overview of models that are available to quantify (parts of) these pathways from human activity to changes in ecosystem functioning and ultimately changes in human well-being. This starts with unravelling the key underlying structures (e.g. marine organisms), processes (e.g. growth and production), and human interactions (e.g. harvesting) of marine ES supply. Furthermore, to assess the effects of human activities on ES supply, a thorough understanding of the cause-effect chains linking human activities to ES is essential.

**Fig. 1:**
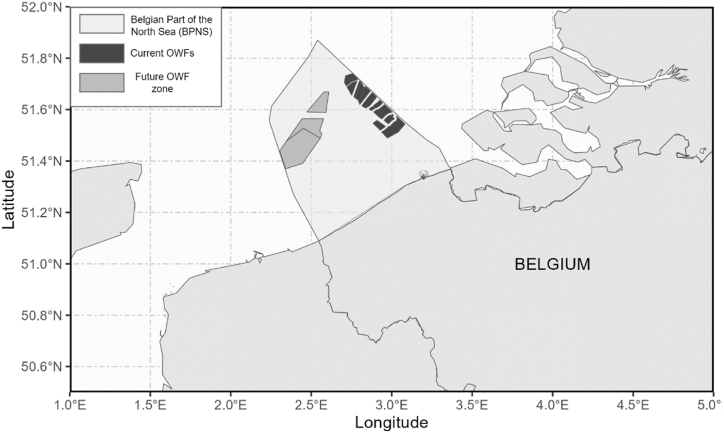
Current and future offshore wind farms (OWF) in the Belgian Part of the North Sea (BPNS).

Therefore, the objective of this research is to propose a method of systematically mapping how marine ecosystems support ES, and to link these with human activities, as a basis for selecting models and indicators. Thus, this research aims to support the transition from qualitative and semi-quantitative to quantitative marine ES assessments. Besides describing a methodological approach for conceptually modelling ES supply to support the selection of models, this research also provides a holistic overview of the local to regional impacts of the presence of an OWF on temperate, shallow-shelf ecosystems, specifically the Belgian part of the North Sea (BPNS) (Fig. 1). In the Belgian Marine Spatial plan, the area reserved for OWFs is planned to increase threefold in the period 2020–2026 [25]. As one of the fastest growing marine activities [26], it is crucial to have a complete picture of the impact of OWFs on ES supply, which will help in the development of sustainable marine spatial planning.

## 2 Approach overview

**Fig. 2:**
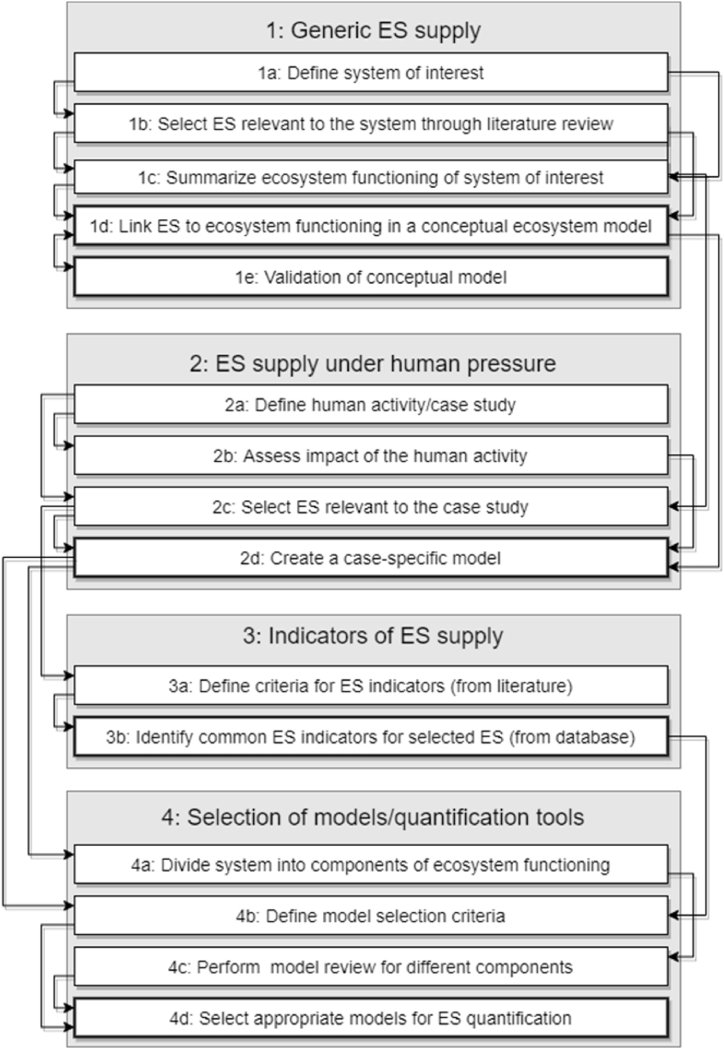
4-tiered method used to holistically map Ecosystem Services (ES) supply and to provide knowledge about which models should be selected to quantify changes in the supply of ES.

A 4-tiered, integrated method to map ES supply and select models for ES quantification is proposed (Fig. 2). The first step is creating an overview of the mechanisms of ES supply in a generic sense, and mapping them accordingly. This can be done for a specific geographical area, such as the Southern North Sea, or a specific habitat type, like a bivalve reef [4]. Existing conceptual models can be used and adapted accordingly to save time. By first creating a generic, fundamental conceptual model on ecosystem functioning, this model can serve as the knowledge base for any case study with a human activity. In the second step, the human activity of interest or related presure is defined, and the conceptual ES model is linked to the activity of interest, including a selection of relevant ES, i.e. not all ES as defined in the geographical system under study are relevant to be assessed quantitatively for a specific activity. This more targeted model allows for the identification of important structures and processes in ES supply. In the third step, indicators relevant for the ES under study are identified and subjected to a set of selection criteria based on studies that evaluate potential criteria, to select appropriate indicators in a transparent process [27]. For the indicators themselves, existing ES indicator databases can be consulted [28]. However, it is very important to critically assess the indicators to ensure their relevance to both the system of interest and the studied human activity. In the fourth step, the knowledge gained in the previous steps results in the development of criteria for the model selection process. For each ES or ecosystem component (a functional part of the system, e.g. the filter feeding community), existing models were reviewed and scored on a set of criteria.

## 3 Case study application

### 3.1 Selection of relevant ES (1a-b)

The 4-tiered approach was applied to the Belgian Part of the North Sea (BPNS) and the results are representative for shallow sub-tidal areas in temperate regions. A list of ES relevant for the BPNS was made by following the Common International Classification of Ecosystem Services (CICES) [29] and performing a literature search focusing on published and grey literature studying “ecosystem services” or “ecosystem functioning” (used as search words) in shallow subtidal areas on temperate continental shelves. A total of 15 services were found to be supplied in the BPNS and similar temperate, shallow shelf systems (Table 1). Among these, six were provisioning services, five regulating services, and four cultural services. This list of relevant ES was ranked by a wide range of stakeholders in the Belgian marine environment, to gain a better understanding of their perceptions of, and preferences for, the services supplied in the BPNS [30]. The top 10 ES from this ranking exercise is used as indication that these services held societal relevance. Based on the ranking by stakeholders, the regulating services were all valued in the top ten, except for *pest control*, which was only added to the list of relevant ES for the BPNS after the meeting with the stakeholders. Of the provisioning services, only *farmed aquatic plants* was not in the top ten and was therefore not included in the list of ES deemed societally relevant. The stakeholders did not select any cultural services in the top ten. However, this could be because the tourism industry and the local inhabitants of coastal regions were not represented at the workshop. Thus, we included *recreation* and *aesthetic value* in the list of societally relevant services based on our literature review for the initial ES list.

**Table 1:** Ecosystem Services (ES) relevant for the Belgian Part of the North Sea (BPNS) included in the initial list with their Common International Classification of Ecosystem Services (CICES) equivalents and whether the service is included in the top-10 of the stakeholder ranking, as an indication of societal relevance. *Service not presented to stakeholders. **Service not selected by stakeholders.

ES type	CICES Group	CICES Class	ES name	Top-10 stakeholders?	References
Provisioning	Cultivated aquatic plants for nutrition, materials or energy	Plants cultivated by in-situ aquaculture grown for nutritional purposes	Farmed aquatic plants (for food, materials and energy)		Causon & Gill, 2018 [36]; UNITED project (Offshore Wind and Flat Oyster Aquaculture & Restoration in Belgium, n.d.) [37]
		Fibers and other materials from in-situ aquaculture for direct use or processing (excluding genetic materials)		No	
		Plants cultivated by in-situ aquaculture grown as an energy source			
	Reared aquatic animals for nutrition, materials or energy	Animals reared by in-situ aquaculture for nutritional purposes	Farmed aquatic animals (for food, materials and energy)		Causon & Gill, 2018 [36]; UNITED project (Offshore Wind and Flat Oyster Aquaculture & Restoration in Belgium, n.d.) [37]; EDULIS project (Offshore Mussel culture in Wind Farms | BLUEGENT, n.d.) [38]
		Fibers and other materials from animals grown by in-situ aquaculture for direct use or processing (excluding genetic materials)		Yes	
		Animals reared by in-situ aquaculture as an energy source			
	Wild animals (terrestrial and aquatic) for nutrition, materials or energy	Wild animals (terrestrial and aquatic) used for nutritional purposes	Wild aquatic animals (for food, materials and energy)		Causon & Gill, 2018 [36]; Vogel et al., 2018 [39]; Hooper et al., 2017 [40]; Busch et al., 2011 [41]; Papathanasopoulou et al., 2015 [42]
		Fibers and other materials from wild animals for direct use or processing (excluding genetic materials)		Yes	
		Wild animals (terrestrial and aquatic) used as a source of energy			
	Mineral substances used for nutrition, materials or energy	Mineral substances used for material purposes	Sand and other minerals	Yes	Degrendele & Vandenreyken, 2017 [43]
	Other aqueous ecosystem outputs	Other	Surface for navigation	Yes	Vogel et al., 2018 [39]; Neyts et al., 2015 [44]
	Non-mineral substances or ecosystem properties used for nutrition, materials or energy	Wind energy	Renewable offshore energy	Yes	Vogel et al., 2018 [39]; Busch et al., 2011 [41]
Regulating	Mediation of wastes or toxic substances of anthropogenic origin by living processes	Bioremediation by microorganisms, algae, plants, and animals	Mediation of wastes		Causon & Gill, 2018 [36]; Volkenborn et al., 2017 [45]; Lindahl et al., 2005 [46]; Braeckman et al., 2010 [47]; Vogel et al., 2018 [39]; Hooper et al., 2017 [40]; Papathanasopoulou et al., 2015 [42]
		Filtration/sequestration/storage/accumulation by micro-organisms, algae, plants, and animals		Yes	
	Mediation of waste, toxins and other nuisances by non-living processes	Mediation by other chemical or physical means (e.g. via Filtration, sequestration, storage or accumulation)			
	Lifecycle maintenance, habitat and gene pool protection	Maintaining nursery populations and habitats (Including gene pool protection)	Nursery and habitat maintenance	Yes	Causon & Gill, 2018 [36]; Degraer et al., 2008 [48]; Vogel et al., 2018 [39]; Hooper et al., 2017 [40]
	Atmospheric composition and conditions	Regulation of chemical composition of atmosphere and oceans	Climate regulation	Yes	Causon & Gill, 2018 [36]; Hooper et al., 2017 [40]
	Regulation of baseline flows and extreme events	Hydrological cycle and water flow regulation (including flood control and coastal protection)	Coastal protection	Yes	Coastbusters project, 2018; Hooper et al., 2017 [40]
	Pest and disease control	Pest control	Pest control	No*	Van der Biest et al., 2017 [80]; Smaal et al., 2019 [49]; Slavik et al., 2019 [50]; Lancelot et al., 2005 [51]
Cultural	Physical and experiential interactions with the natural environment	Characteristics of living systems that enable activities promoting health, recuperation or enjoyment through active or immersive interactions	Recreation	No**	Causon & Gill, 2018 [36]; Degraer et al., 2019 [9]; Vogel et al., 2018 [39]; Hooper et al., 2017 [40]; Van der Biest et al., 2017 [80]
		Characteristics of living systems that enable activities promoting health, recuperation or enjoyment through passive or observational interactions			
	Intellectual and representative interactions with the natural environment	Characteristics of living systems that enable aesthetic experiences	Aesthetic value	No**	Vogel et al., 2018 [39]; Hooper et al., 2017 [40]; Busch et al., 2011 [41]
		Characteristics of living systems that enable scientific investigation or the creation of traditional ecological knowledge	Scientific research	No	Vogel et al., 2018 [39]; Hooper et al., 2017 [40]
		Characteristics of living systems that are resonant in terms of culture or heritage	Cultural heritage	No	Vogel et al., 2018 [39]; Causon & Gill, 2018 [36]; Busch et al., 2011 [41]

### 3.2 Generic conceptual ecosystem model (1c-e)

**Fig. 3:**
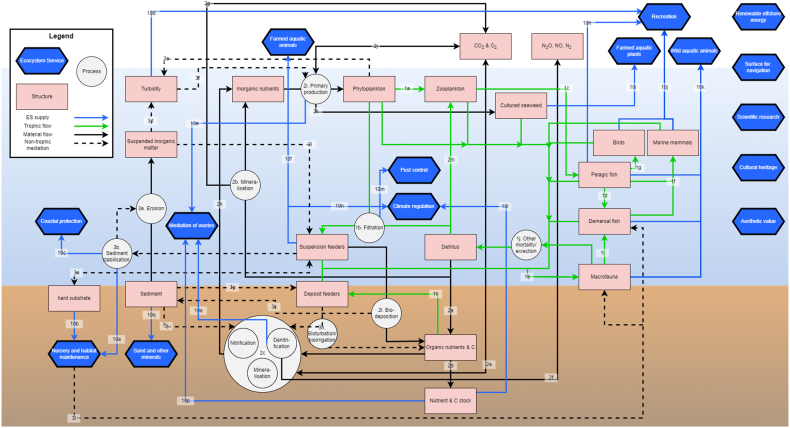
Generic conceptual ecosystem services (ES) model for the BPNS, including inputs from several experts. Blue hexagons, red boxes, and white circles represent ES, structures and processes, respectively. Blue arrows represent ES supply, green arrows represent trophic flows, black arrows material flows and dotted arrows represent non-trophic mediations. The model is non-directional and summarized ecological knowledge on ES supply. Each link is labelled with the ID used in the evidence library (see Supplementary Material 1).

For the 15 services found to be ecologically relevant in the BPNS and similar systems, we constructed a conceptual model detailing the major structures and processes through which these services are supplied (Fig. 3). Structures (red rectangles) are defined to be all stocks naturally present in the ecosystem, e.g., species biomass, nutrient concentration, and physical structures, but could also refer to man-made constructions that have become an integral part of ecosystem functioning (e.g. wind turbine foundation). Processes (white circles) refer to changes in the structures of the biophysical system, as well as influences of one structure on another. These structures and processes supply the relevant ES (blue arrows leading to blue hexagons). The structures and processes are linked by material flows, trophic flows, and influences. Material flows (solid black arrows) indicate the movement and/or transformation of non-living materials from one group or process to another, such as the burial of *organic nutrients and carbon* in the *nutrient and carbon stock* in the sediment. Trophic flows (solid green arrows) indicate the consumption of one living (trophic) group by another, such as the consumption of phytoplankton by zooplankton, as well as the mortality of all living groups to detritus. Finally, influences (dashed black arrows) indicate that one structure or process affects another without the presence of a flow of material or biomass, e.g. the (negative) effect of increased turbidity on primary production. The exclusion of forcing variables (from outside the system under study) means the model is focused solely on the behaviour generated within the system.

The construction of the conceptual model was based on the fourth step of the method by Van der Biest et al. (2020) [31] for setting ecologically sensible objectives, in which the processes that contribute to ES development, maintenance or delivery are identified for each habitat and ES of interest. Ecosystem functioning of sediments and the water column of temperate shallow shelf systems was derived from Van der Biest et al. (2017) [80], where a comprehensive overview of services supplied in the Belgian marine and coastal areas is provided. In addition, we also used other sources to complete specific parts of the conceptual model as documented in an evidence library, as per Olander et al. (2018) [4]. The evidence library can be found in Supplementary Material 1, alongside an explanation of the information stored in it and the assessment of the strength of the evidence.

The generic conceptual model can be broadly summarized in three domains: 1) The food web and ES associated with it, on the right side of the figure. The food web is comprised of the predator-prey relations from low trophic levels (phytoplankton, zooplankton) to higher levels (fish, birds, marine mammals). All organismal groups feed into the detritus group through excretion and mortality. 2) The nutrient cycle, taking place mostly in and on the sediment, on the bottom of the figure. Here, organic carbon and other nutrients from the detritus group enter the sediment and can be remediated in the sediment through multiple processes. The rates of these processes are influenced by the activity of deposit feeders through bioirrigation and bioturbation. 3) Physical processes, in the sediment, on the left side of the figure. Processes such as deposition, erosion and sediment stabilisation can be influenced by biotic groups such as suspension feeders, which can affect the ES supply of the sediment.

We divided the benthic biota into suspension feeders (e.g. mussels and anemones, epibenthic organisms, mostly on hard substrate), deposit feeders (e.g. polychaetes, endobenthic organisms, mostly on soft substrate) and macrofauna (e.g. crabs and echinoderms, mostly large-bodied, mobile predators). It is important to note that this distinction is a highly simplified view of benthic ecology. For instance, suspension feeders can be found both on hard and soft substrates. The ES identified as being provided by the BPNS system as a whole and/or not directly related to specific ecosystem functions, are pictured on the right side of the model, with no direct ES flows.

The conceptual model was validated with expert opinions through interview sessions with a total of five scientific experts in the field of ES assessment, benthic functioning, and trophic interactions (Supplementary Material 2). In these interviews, the experts were asked to follow cause-effect chains through the conceptual model to identify missing or irrelevant components and faulty assumptions. The draft model was updated after carefully considering the inputs from the experts, resulting in the final conceptual model of ES supply in the BPNS or areas with similar conditions.

### 3.3 Local to regional impact assessment (2b)

Reviewing literature, we identified changes to ecosystem components, structures and processes [32] that are of importance in the supply of marine ES, resulting from the presence of OWFs in the BPNS and similar systems. While impact tends to have a negative connotation, it is possible that changes in certain variables result in an increased supply of ES. These qualitative changes in environmental variables were extracted from available literature and reports that discuss results of environmental monitoring programs around OWFs in the BPNS [33]. The recorded changes often varied over time since the intervention and were not always directly linkable to OWF presence. Nevertheless, multiple studies identified several key impacts, which can be attributed to different aspects of the introduction of OWFs:

1. Introduction of hard substrate:
  a. Introduction of fouling communities (i.e. a strong increase in the local biomass of hard substrate species such as *Mytilus edulis, Metridium senile* and *Jassa herdmani*)*.*
  b. Increase in total benthic habitat, biomass, density, biodiversity, and community composition.
  c. Benthic total organic matter content increases due to an increased flux of biodeposits from the fouling community.
2. Hydrodynamic changes:
  a. Sediment fining (median grain size, fine sediment fraction) resulting from changes in hydrodynamic conditions and increase flux of biodeposits from the fouling community.
3. Introduction of cables and their associated electromagnetic fields, the impacts of which were not yet monitored in the BPNS.
4. Exclusion of fisheries:
  a. Changes in demersal fish species density (e.g. increase in *Pleuronectes platessa*).

### 3.4 Case-specific impact model (2c-d)

To adapt the generic model to a specific case study, we constructed a conceptual model of the effects of OWF presence on the functioning of the biophysical system and, subsequently, on ES supply. To do so, we integrated the conceptual ecosystem model (Section 3.2) and the local to regional impact assessment (Section 3.3), following the approach of Olander et al. (2018) [4]. We expand on Olander et al. (2018) [4] by using our understanding of ES supply from the generic conceptual ecosystem model to construct the case-specific impact model.

**Fig. 4:**
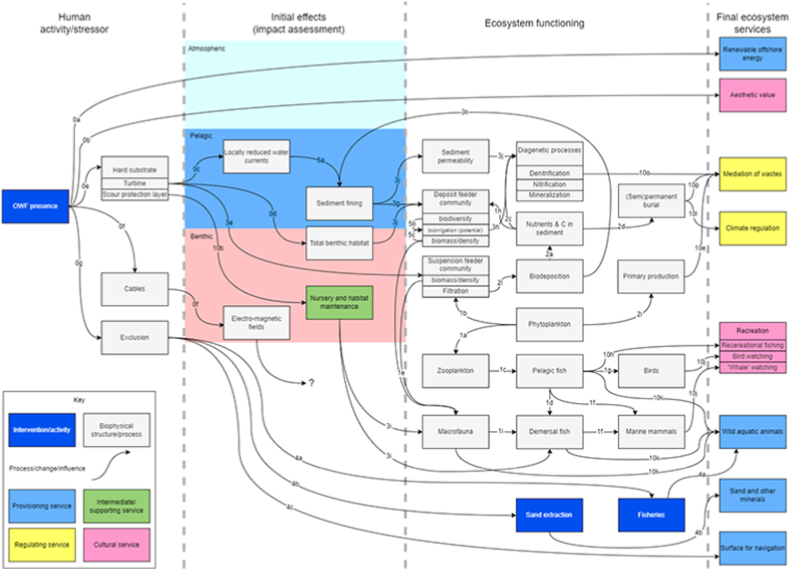
Conceptual case-specific impact model of Offshore Wind Farms (OWF) presence on Ecosystem Services (ES) supply. The pathways are divided into the human activity/pressure of interest, the initial effects, ecosystem functioning and supporting services, and final ES. The labels of the linkages refer to the code in the evidence library. The white boxes, connected by arrows, represent structures and processes in the cause-effect chain(s) from the initial human activity to the supply of ES.

Not all the services that were found to be ecologically and societally relevant in the area under study were included in this model. Rather, we only include ES for which changes in (the structures and processes underlying) supply due to the presence of OWFs were found in the local to regional impact assessment (3.3). From the list of twelve ES deemed societally relevant (Table 1), nine were found to be impacted by the presence of an OWF, i.e. we identified research that shows changes in the supply of these ES or the structures and processes underlying it (Evidence library in Supplementary Material 1). ES that were not found to be impacted by OWF presence were *farmed aquatic animals, coastal protection* and *pest control*. For the other services, we connected the variables from the local to regional impact assessment studies (Section 3.2 Local/regional impact assessment) to variables in the generic conceptual ecosystem model. We only included links for which the evidence was moderate to strong (Supplementary Material 1), because the purpose of this work was to develop a support tool for quantitative assessments: If evidence for interaction with OWF and other human activities is insufficient, quantification will most likely not be possible. We mapped the pathways from the initial intervention through ecosystem functioning and supporting services, to final ES, to give a holistic overview of the pathways in which OWF presence influences ES supply. Fig. 4 presents the case-specific impact model of OWF presence to the supply of ES, where the labels refer to the evidence library (Supplementary Material 1). The impacts in the model are depicted directionally from left to right, starting at the initial human activity, and ending at the affected ES, with arrows representing cause-effect chains between relevant structures and processes. These correspond to the (non-directional) interrelations in the generic ecosystem services conceptual model (Section 3.2). Broadly speaking, the top-half of the model represents the physicochemical pathways, and the bottom-half represents the trophic pathways. In this conceptual model, other human activities that interact with OWF are also depicted. Although these activities have their own impacts on the marine environment [32] and, subsequently, on ES supply, this is not considered here.

Similar to the generic conceptual ecosystem model, the case-specific impact model does not depict external pressures that may be important in ES supply but focuses on the internal dynamics as influenced by OWF presence. It is important to note that the generic ecosystem model and case-specific impact model do not explicitly depict spatial dimensions, but, as Olander et al. (2018) [4] state, “they can help identify where spatial dynamics will be critical, for example where distance to a resource or exposure can affect human use or impact”. The model presents a holistic overview of how an OWF may affect the local natural system and socio-economic well-being and provides an overview of the parts of the biophysical system that need to be considered for quantitative assessments.

### 3.5 ES indicators (3a-b)

For the prioritised ES that were included in the case-specific impact model (Table 1), indicators were selected that can best be used to quantify and assess those services (Table 2). For the indicator selection, we consulted the structured marine ES indicator pool by Von Thenen et al. (2020) [28]. For each service, we assessed which indicator(s) met the nine key criteria proposed by Van Oudenhoven et al. (2018) [27]. Supplementary Material 3 gives an overview of how each of these criteria is met in this research. In some cases, multiple indicators were found to be relevant and useful for one ES. The selected indicators were used to select appropriate models for the relevant ES.

**Table 2:** Indicators selected from the indicator database [28] for each of the selected Ecosystem Service.

Ecosystem service	Selected indicator(s)	Units
Renewable offshore energy	- Energy production	- TWh/year
Aesthetic value	- Visual impact of artificial structures from the shore	- Impact factor (qualitative indicator)
Mediation of wastes	- Denitrification - Burial	- tonnes N/year - tonnes N/year
Climate regulation	- Carbon sequestration rate	- tonnes C/year
Recreation	- Production (recreational fishing) - Biomass (*potential* supply of recreational fishing) - Abundance (and diversity) of observed species (wildlife watching)	- tonnes/year - tonnes
Wild aquatic animals	- Production - Biomass (indicator of *potential* supply)	- tonnes/year - tonnes
Sand and other minerals	- Volume of sand available for extraction	- m^3^
Surface for navigation	- Length of shipping lanes - Tonne-kilometres of shipping traffic	- Km - tonne-kilometre
Nursery and habitat maintenance	- Species richness - Shannon diversity D	- n species - bits/ind

### 3.6 Model selection (4a-d)

A literature review was performed on available quantitative and semi-quantitative models for the ES identified to be relevant in the case study (Fig. 4). *Renewable offshore energy* was left out of the review because the supply of this service does not interact with the biophysical system or the users of the system. Furthermore, we did not perform the review for *nursery and habitat maintenance*, as this is a supporting service that should be incorporated into models for the ES it supports (*wild aquatic animals*, *recreation*). Our focus was on mechanistic models, as these describe in a detailed and specific manner the (causal) relationships between the physical structures and processes in the system. For some ecosystem components, we also broadened the scope and reviewed empirical models, such as regression models or other statistical relationships, multi-criteria models and socio-ecological models (e.g. travel cost and choice models). This was mainly due to unavailability of mechanistic models. These models do not, however, explain the ecological mechanisms driving ES supply, but rather describe relationships based on real-world observations.

Besides searching for models that explicitly depict ES [34,35], our searches also included keywords related to ecosystem functioning (e.g. “nutrient cycling” rather than “mediation of wastes”). Additionally, we relied on review papers that summarized different models of the same subject (e.g. Hyder et al. (2015) [24] for models of fisheries and the food web). A total of 85 models was identified for different parts of the ecosystem (Supplementary Material 4). For each model, we identified:

- General model information: which ES and ecosystem compartment was/were modelled? For what geographical area has the model been developed? What type of model is it (mechanistic/empirical, ecosystem/single-species, individual-based/biomass-based, score-based/quantitative, etc.)?
- For models that were identified in review articles, we also noted the initial reference source.

To select the most appropriate models to be used in quantitative ES supply modelling, we defined four criteria:

1. The model contains variables that are *relevant for the changes identified in the impact assessment.* For this, we identified whether the selected indicators (Section 2.5) were represented in the models to link between the impact of a specific human activity (OWF) and the supply of relevant ES. If there is no overlap of one or more variables, than the model is not deemed suitable. For example, the biofouling communities on OWFs increase supply of organic matter to the benthos, so benthic nutrient cycling models including organic matter input as variable are preferable.
2. The model represents *relevant pathways of the conceptual case-specific impact model.* For this, we extracted detailed model information: what is the specific content and scope of the model (e.g. nitrogen and phosphorus process rates, fish community dynamics, shellfish growth model, etc.)? Which processes are explicitly modelled? How is each component/ES represented in the model (e.g. fishing, bivalve biomass)? This was then compared to the structures, processes and services in the conceptual case-specific impact model, to identify overlap with the model.
3. Information on *model complexity*: which dimensions are modelled? Is it temporally and/or spatially explicit? For each model we qualitatively assess the complexity (low/medium/high) based on these factors, as well as on how many structures and processes are represented relative to our needs. Models with a high complexity were not selected because it limits the models' usefulness in a broadly applicable ES assessment tool.
4. Lastly, we assessed if the model or its outputs can be linked to other ES/ecosystem compartments, and therefore *allows for the analysis of trade-offs between ES.* Preference is given to models that allow for the assessment of interactions between ES (e.g. food web model) or with common input parameters (e.g. benthic community as an input parameter for the model of water quality regulation and for the food web model).

The model review resulted in a classification of different models for the researched ES, and a subsequent selection of the most suitable models. The whole list of ecosystem models identified in the review can be found in Supplementary Material 4. In Table 3, we present our findings on the most suitable models to be used in a holistic and quantitative ES assessment of the case study. For each ES, information is provided on the four criteria set up to select models. For the services that do not interact with the ecological system, e.g. *aesthetic value* and *sand and other minerals*, links with other services or ecosystem components were not identified.

**Table 3:** Information on how the selected models for each Ecosystem Service (ES) meet the four criteria set up to select models for a quantitative assessment.

General information				1: Impact assessment	2: Part of conceptual model		3: Complexity	4: Linking
(Ecosystem) component	ES	Selected model	Reference	Model variables	Description	Indicators	High/Medium/Low	Linked to:
Nutrient cycle in sediment	Mediation of wastes	Regression model	Toussaint et al., 2021 [52]	Denitrification, % organic matter, % fine sediment, irrigation potential, bio-irrigation rate	Regressions between sediment parameters and nutrient processes	Nitrogen removed (tonnes N/km^2^/year)	Low	Community composition
	Climate regulation	Accumulation rate formula	Middelburg, 2019 [53]	C accumulation, sediment accumulation rate, porosity, dry density, C deposition	Formula of carbon storage resulting from an increased flux	Carbon stored (tonnes C/km^2^/year)	Low	C deposition by filter feeders, community composition
Food web	Wild aquatic animals	EMBENS Ecopath model	Pint et al., 2021 [54]	Biomass, production, consumption, landings, discards, diet	Full food web and fisheries model, with a.o. biomass and harvests	Biomass (tonnes/km^2^) Landings (tonnes/km^2^/year)	Medium/high	Community composition, possibly biochemical, socio-economic models, habitat preferences, …
(Biofouling) Filter feeders	Mediation of wastes, climate regulation	COAWST model (output)	Ivanov et al., 2021 [55]	Mussel density, C deposition, filtration, chlorophyll-α concentration	Modelling output of a coupled hydrodynamic and biogeochemical model	–	Low/medium	Water column and sediment processes
Sediment resources	Sand and other minerals	Spatial conflict analysis	Van Lancker et al., 2019 [56]	Holocene sediment layer thickness, available sand (with reference levels)	Spatial overlap of concession zone and sediment layers	Volume of sand available for extraction (m^3^)	Low	–
Recreation	Recreation	Biodiversity indicators	–	Bird biodiversity, bird abundance, willingness-to-pay, travel cost	Model linking biodiversity and abundance indicators of birds to socio-economic value	Number of species (birds) Abundance (birds)	Low	Bird abundance in food web
Navigation	Surface for navigation	Spatial conflict analysis	–	Shipping lanes, shipping traffic	Analysis of added costs of blocking and rerouting marine traffic	Length of shipping lanes (km) Tonne-kilometres of shipping traffic (tonne-kilometre)	Low	–
Aesthetic value	Aesthetic value	Visual impact model	Maslov et al., 2017 [57]	Distance to shore, aesthetic impact index	Model to calculate the impact of structures on the seascape	Visual impact score (0–1)	Low	–

## 4 Discussion

This research offers a useful framework to disentangle the structures and processes that underly the supply of ES and to select models to quantify ES changes due to human pressures. The framework developed here could also be an efficient and consistent support tool for marine spatial planning as it allows users to gain insight into the interactions between human activities and the marine environment, and to identify how societal benefits acrued from the supply of ES are distributed among stakeholders [4]. The conceptual ecosystem model also serves as a summary of our understanding of ES supply in the BPNS and similar ecosystems. The model represents the mechanisms in the marine ecosystem by which ES are supplied in a condensed and integrated manner and provides insight into which structures and processes are most important in the supply of ES. It is novel in depicting the links between a wide range of ecosystem functions and services in a comprehensive overview. The compiled evidence library (Supplementary Material 1) offers a theoretical underpinning of the conceptual model, and serves as a useful reference work of ecosystem functioning. This general knowledge base can be used to gain insight into the effect of any human activity on ES supply, similar to what was done here for the presence of OWFs in the BPNS.

The reasons to create conceptual ES models for case studies are plentiful, and the results can help to integrate ES assessments into decision making. Conceptual models of ES supply can facilitate discussion between stakeholders with different backgrounds; it can help to align experts and stakeholders on ecosystem management, by identifying priorities and linking them to human interventions in a transparent and systematic way. They also provide evidence-based qualitative assessments of the implications for ES of certain potential interventions. Furthermore, they may help identify gaps in system understanding which can inspire new research. Conceptual ES models can also be used to identify the most relevant metrics of socio-economic value and provide a foundation for quantitative assessments. For example, in the case of OWFs, we identified indicators that best capture changes in the affected ES [4]. An aspect that this research does not reflect on is the different temporal and spatial scales on which these changes act, which is important for assessing the magnitude of changes in ES supply when moving towards quantification [58].

This is the first study to holistically map the cause-effect chains starting from the presence of OWF to the supply of ES using (mostly) empirical systems understanding and existing indicators. Our findings show that OWF presence affects both the biotic and abiotic components of the marine environment, and that these components interact with eachother intensely. We found that OWF presence affects ES supply in many ways, from direct effects (e.g. the impact on *aesthetic value*) to longer cause-effect chains through the marine environment (e.g. changes in *recreation* and food supplied by *wild aquatic animals* as a result of changes in the lower foodweb). Our work builds on research that identified the many impacts that OWF installation and presence has on the marine environment [36,59]. These findings highlight the importance of comprehensive environmental impact assessment when planning and managing activities such as offshore energy. This research also highlights the knowledge gaps regarding the effects of OWFs, such as the environmental responses to magnetic fields caused by underwater power cables, as well as the magnitude and duration of the changes in the foodweb.

Other studies that synthesize the effects of human activities such as OWF presence on ES supply, on which this research builds, often take a less holistic approach. For instance, studies that focus on specific compartments of the ecosystem rather than the full range of affected structures and processes, e.g. changes in epibenthic community due to OWF [36,60]; or studies that provide a holistic overview of the services affected by OWFs but only identify impacts and do not describe pathways supplying ES [32,[39], [40], [41]]. Studies that represent the pathways from the human impacts to ES supply are often qualitative and expert-based [31,42]. These studies make these necessary trade-offs because of the inherent complexity of assessing ecosystem services comprehensively and quantitatively. In this study, we expand on these examples by holistically mapping the cause-effect pathways and selecting appropriate indicators and models that represent these pathways, allowing for quantification of ES supply.

The four-tiered approach proposed here led to a selection of models and other tools to quantify ES of varying complexity, which allows those interested in quantifying changes in ES supply to select tools that best fit their needs. The overview of ecosystem functioning models (Table 3) may serve as a useful reference for researchers aiming to quantify marine ES, either as a result of OWFs or another offshore human impact. For each ES, we identified tools that capture (subtle) changes in ES supply in the most simple computational, yet scientifically underpinned manner possible, to optimize ease of use in ES assessments. However, this was difficult for some ES, because of the inherent complexity of some parts of the ecosystem. For example, to quantify changes in the structures and processes that make up the marine foodweb, relatively complex models of trophic interactions were selected as the best way to capture the effect of human activities on ES related to the foodweb. Foodweb modelling is inherently complex and time-consuming, making it challenging to quantify changes in these services [61]. However, we still opt for this method because it is one of the few identified methods that can be used to make meaningful statements regarding foodweb-related ES. For other services, e.g. nutrient cycling, we selected approaches that do not mechanistically model ecosystem processes, but rather quantify ecosystem functioning using empirical relationships, such as regression models. Although these models need to be interpreted with caution, they offer a useful tool to translate the complexity of ecosystem functioning to an ES context. Given the scarcity of existing holistic quantitative ES models for the marine environment, using and adapting existing models has the potential to greatly advance marine ES assessments.

The current research represents an intermediate step between qualitative and quantitative assessments of ES supply. It is useful for gaining an understanding of ES supply and communicating ES studies to stakeholders. However, more work is needed to apply the selected modelling tools, collect the necessary biophysical data and to undertake the quantitative assessment of ES supply, applying it also in other geographical areas. This work focused on the supply side of the ES cascade, which means the socio-economical consequences of the researched changes in ES supply are not made explicit. To increase the usefulness of such models, there is a need to link the output of ES supply models to socio-economic scenarios [61]. To do so would require a more interdisciplinary approach than was feasible in this study. The validation of the model was done through expert opinion. Though this is a valid method of validation for such conceptual models, it not the most desirable. Preferably, validation would be done with quantitative models or measurements, or the application of the model to different case studies. This falls outside of the scope of this study. Integration of ESA with other disciplines, such as Life Cycle Assessment (LCA) and other impact assessment methodologies, is beneficial to assess the full cause-effect chain because ESA is an ecosystem-based approach that cannot quantify the impacts of human activities at different scales [11]. Currently the most used approach for integrating impact assessments with an ES approach in the marine environment is to map human activities, map habitat distribution, score how human activities impact habitats and score how habitats supply ES (e.g. Refs. [[62], [63], [64], [65], [66], [67]]). When linking this in a cause-effect chain, as done in this study, the consequences of different management scenarios on ES supply can be (semi-)quantified. The advantage of this approach is that the effect of multiple human activities on a wide range of ES can be assessed, relatively quickly. However, this is mostly score-based, and does not quantitatively represent the structures and processes supplying ES. Culhane et al. (2019) [69] performed such an analysis for a higher resolution of habitat (dubbed ‘ecosystem component’), however, it is still a score-based approach that does not explicitly consider the biophysical reality and is reliant on available expert judgement. Furthermore, it is difficult to integrate scores to make statements on synergistic or antagonistic effects on ES supply. In terrestrial systems, linking environmental impact assessments with an ES approach is more developed and has also been applied quantitatively, beyond score-based assessments [70]. This is more challenging in the aquatic environment because of the three dimensionality and multi-use of space [71]. The only studies that do so are ecotoxicological studies [[72], [73], [7][4]]], or studies that quantify aquatic ES supply without quantifying the effects of specific human activities [75]. This is due to the single-pressure nature of ecotoxicological impact and the well-studied effects on fixed ecological structures (organisms). These studies all start by mapping the cause-effect chains of the pressure. For more complex human pressures, i.e. those that impact the system in multiple pathways, quantification is harder to perform. Compared to the widely used DPSIR framework, the approach proposed in this work puts more emphasis on the socio-economic outcomes in terms of ES, in line with the newer DAPSI(W)R(M) framework [3]. Altogether, there is a large knowledge gap in quantifying the linkage between environmental impact assessment and ES assessment [11], and the current work proposes a method to identify which parts of the biophysical structures and processes need to be considered to assess ES quantitatively. Given that the mapping of ES is mandatory for EU member states, enhancing our ability to perform precise and accurate ES assessments is vital [76].

Environmental impact assessment studies and the studies on changes of ES due to human activities can benefit from each other. The ES framework provides an opportunity to define clear ecological objectives to impact assessments [77]. Also, ES assessment can serve as good resilience descriptors for the state of (marine) ecosystems under diverse human pressures that change in time and space [78]. Impact assessment and ESA are integrated in several modelling frameworks, most prominently by using the Integrated Valuation of Ecosystem Services and Tradeoffs (InVEST) modelling toolbox [79]. However, these models are limited in the integration of ecological processes such as trophic interactions, most often linking a single-species population to a habitat, and calculating the risk posed by human interventions to that habitat. Using and combining existing ecosystem functioning models is a promising way forward for the integration of environmental impact assessments and ESAs, and more inclusive and quantitative assessments of ES supply.

To safeguard the supply of marine ES under increasing human activity at sea, an ecosystem-based approach is vital, and should be central in the development of the blue economy. The conceptual models presented in this work can aid the governance of marine ecosystems by providing managers with the tools to view the environment as a producer of goods and services for human well-being. Specifically, this work helps to: (i) further increase sustainability awareness among economic players and provide concrete tools to support the design and implementation of more sustainable, ecosystem-based solutions; (ii) transition to a blue economy where ecosystem-based thinking constitutes the backbone in the development of all marine activities; (iii) reach the goals of the Marine Spatial Plan, i.e. multi-functionality and embedding working-with-nature principles in all future developments.

## 5 Conclusion

This research aimed to develop a systematic method for mapping how marine ecosystems support ES and linking these with human activities, as a basis for selecting models and indicators that can be used to quantitatively assess marine ES. It supports the transition from qualitative and semi-quantitative to quantitative marine ES assessments. In order to accurately judge the state of the marine system – and the ES it supplies – it is inadequate to merely assess fixed ecosystem parameters and the distribution and state of habitats: One must also gain an understanding of the processes underlying ecosystem functioning and ES. Mapping the pathways of human impacts on ES supply enables researchers to objectively evaluate the influence of human-induced pressures, e.g. from Blue Economy initiatives, on ES supply and offers new perspectives on the tools used to assess changes in ES supply. This study offers a useful method to select modelling tools effectively and transparently, for fully quantitative ES assessments in the marine environment.

## Author contribution statement

Van de Pol, Lennert: Conceived and designed the experiments; Performed the experiments; Analyzed and interpreted the data; Wrote the paper.

Van der Biest, Katrien: Conceived and designed the experiments; Performed the experiments; Analyzed and interpreted the data; Wrote the paper.

Taelman, Sue Ellen: Conceived and designed the experiments; Wrote the paper.

De Luca Peña, Laura: Conceived and designed the experiments; Wrote the paper.

Everaert, Gert: Conceived and designed the experiments; Wrote the paper.

Hernandez, Simon: Conceived and designed the experiments; Performed the experiments; Wrote the paper.

Culhane, Fiona: Contributed reagents, materials, analysis tools or data; Wrote the paper.

Borja, Angel: Contributed reagents, materials, analysis tools or data; Wrote the paper.

Heymans, Johanna J.: Contributed reagents, materials, analysis tools or data; Wrote the paper.

Van Hoey, Gert: Contributed reagents, materials, analysis tools or data; Wrote the paper.

Vanaverbeke, Jan: Contributed reagents, materials, analysis tools or data; Wrote the paper.

Meire, Patrick: Conceived and designed the experiments; Wrote the paper.

## Funding statement

This work was supported by Agentschap Innoveren en Ondernemen and powered by De Blauwe Cluster - the spearhead cluster for blue growth in Flanders (The Blue Cluster) - within the framework of the SUMES project [HBC.2019.2903]. Angel Borja was supported by GES4SEAS project, funded by the European Union under the Horizon Europe program [101059877], JJ Heymans acknowledges funding from the European Union's Horizon 2020 research and innovation programme [101000302].

## Data availability statement

No data was used for the research described in the article.

## Declaration of interest’s statement

The authors declare no competing interests.
