# Supplementary material for: Impacts of human activities on the supply of marine ecosystem services: A conceptual model for offshore wind farms to aid quantitative assessments
Source: Heliyon. 2023 Feb 15;9(3):e13589. doi: 10.1016/j.heliyon.2023.e13589 (PMC9958457; doi:10.1016/j.heliyon.2023.e13589)
Supplement: Multimedia component 1 [file mmc1.docx]

| **# Interview** | **Participant** | **Affiliation** | **Focus** |
| --- | --- | --- | --- |
| 1 | Jan Vanaverbeke | Royal Belgian Institute of Natural Sciences | Benthic functioning |
|  | Gert van Hoey | ILVO |  |
| 2 | Sheila Heymans | European Marine Board, VLIZ | Marine food webs |
|  | Angel Borja | AZTI |  |
| 3 | Fiona Culhane | University of Plymouth | Marine ES supply |
