# Supplementary material for: Impacts of human activities on the supply of marine ecosystem services: A conceptual model for offshore wind farms to aid quantitative assessments
Source: Heliyon. 2023 Feb 15;9(3):e13589. doi: 10.1016/j.heliyon.2023.e13589 (PMC9958457; doi:10.1016/j.heliyon.2023.e13589)
Supplement: Multimedia component 5 [file mmc5.docx]

**Supplementary Material**

1. Evidence libraries of conceptual models, references used, and explanation of evidence library (excel file)
2. Summary of the interviewed experts for the validation of different parts of the generic ecosystem model.

| **# Interview** | **Participant** | **Affiliation** | **Focus** |
| --- | --- | --- | --- |
| 1 | Jan Vanaverbeke | Royal Belgian Institute of Natural Sciences | Benthic functioning |
|  | Gert van Hoey | ILVO |  |
| 2 | Sheila Heymans | European Marine Board, VLIZ | Marine food webs |
|  | Angel Borja | AZTI |  |
| 3 | Fiona Culhane | University of Plymouth | Marine ES supply |

1. **Indicator selection criteria from Van Oudenhoven et al. (2020) and a justification for how these criteria were met in this research.**

| **#** | **Criterion** | **Justification** |
| --- | --- | --- |
| 1 | The indicators’ scientific validity and implementation in relatable decision-making context have been backed up by data or documented evidence. | All indicators come from a peer-reviewed indicator pool (Von Thenen et al., 2020), and are scientifically valid. |
| 2 | The value of the indicator will change if the issue of concern to decision makers changes | Only those services were selected that are known to be affected by the presence of Offshore Windfarms, ensuring that indicators selected for those services change as a consequence of windfarm presence. |
| 3 | The indicators have been assessed and co-selected by relevant scientists, experts and decision makers in a transparent review process. | All indicators come from a peer-reviewed indicator pool (Von Thenen et al., 2020), and were selected in a transparent review process. |
| 4 | The needs and perspectives of decision makers and relevant stakeholders have bene incorporated in defining the purpose, context and type of outputs of the indicators. | Stakeholders’ perspectives were included in the selection of relevant ecosystem services. |
| 5 | The indicators allow for tracking progress towards achieving relevant policy goals or local visions. | Only those services were selected that are known to be affected by the presence of Offshore Windfarms, ensuring that indicators selected for those services can be used to track progress towards policy goals regarding the environmental impact of offshore windfarms. |
| 6 | The understandability of the ES indicator has been tested with relevant actors, ensuring communicability and potential awareness raising. | All indicators come from a peer-reviewed indicator pool (Von Thenen et al., 2020), and are communicable. |
| 7 | The required data and information are available for assessing the indicator at an appropriate temporal and spatial scale and from recognized sources. | By only including environmental impacts that have been (empirically) shown to occur as a consequence of windfarm presence, and by only including interactions in the model for which the evidence is strong, relevant data availability is ensured. |
| 8 | The required process of indicator quantification is affordable and feasible for the responsible institution, and it includes sufficiently frequent monitoring. | By only including environmental impacts that have been (empirically) shown to occur as a consequence of windfarm presence, and by only including interactions in the model for which the evidence is strong, the feasibility of indicator quantification is ensured. |
| 9 | There is an implementation plan, including sufficient time and resources allocated, to develop the indicator, to evaluate it during its use, and to adapt the indicator when required. | The selection process can be revisited throughout the process and sufficient time is dedicated to evaluating the suitability of the indicators. |

1. **Model review results and overview (excel file)**
